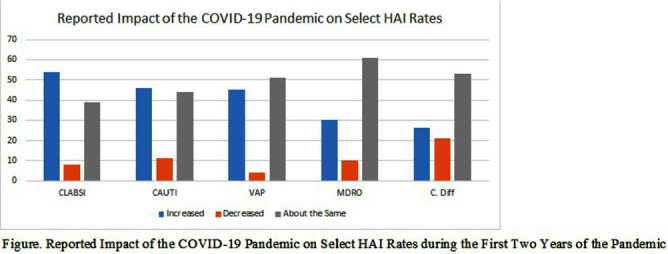# The Impact of COVID-19 on Healthcare-Associated Infections: A Survey of Acute Care Hospitals

**DOI:** 10.1017/ash.2024.110

**Published:** 2024-09-16

**Authors:** Monika Pogorzelska-Maziarz, Julia Kay, Tara Schmidt, Anika Patel, Pamela De Cordova

**Affiliations:** Thomas Jefferson University; Thomas Jefferson University, College of Nursing; Rutgers, the State University of New Jersey

## Abstract

**Background:** The COVID-19 pandemic has placed an enormous strain on the healthcare system, including infection prevention and control. The response to the COVID-19 pandemic required extraordinary resources, which were often diverted from routine infection prevention and control activities and may have contributed to increased rates of HAI in the acute care setting. However, the impact of the COVID-19 pandemic on infection prevention and control departments, including staffing and resources, and on routine infection prevention and control activities is not well-described. The objective of this study was to describe the impact of the COVID-19 pandemic on IPC departments and department response to the pandemic. **Methods:** Between August and December of 2023, we conducted an electronic survey of all acute care facilities participating in the National Healthcare Safety Network. Survey data were analyzed using descriptive statistics. **Results:** Over 594 infection control departments participated in the survey, representing 1,400 NHSN facilities (20% response rate based on number of eligible NHSN facilities). Half of the respondents reported that their hospital experienced increases in the following HAI rates during the first two years of the pandemic: central-line associated bloodstream infections (54%), catheter-associated urinary tract infections (46%) and ventilator associated pneumonia (45%). When asked to identify the top three contributors to increased HAI rates in their facility, respondents cited the following factors: staffing shortages (70%), patient acuity (69%), use of travel nurses (48%), increased device utilization (37%), and reduced bedside acuity (31%). Respondents reported that their department utilized the following actions to decrease these HAI rates: increased rounding and monitoring of IPC procedures (81%), reeducation of frontline staff on IPC policies and procedures (77%), environmental care rounds (69%), monitoring of isolation compliance (66%), HAI Task Force/Committee (57%), nurse-driven catheter removal protocols (53%), and insertion prevention protocols (53%). When asked if the department experienced applied pressure or attempts to influence HAI reporting due to the increase in HAI rates in the facility experienced in the wake of the pandemic, 19% of respondents reported increased pressure from management/C-suite and 7% reported increased pressure from providers. **Conclusion:** The COVID-19 pandemic had a substantial impact on IPC departments in acute care hospitals and had a profound effect on IPC staffing, resources and routine IPC activities. Future work needs to identify best practices and lessons learned from the pandemic to inform future pandemic preparedness.

**Figure:**